# Physiological role of anticipatory cardiorespiratory responses to exercise

**DOI:** 10.14814/phy2.15210

**Published:** 2022-03-05

**Authors:** Tadayoshi Miyamoto, Daisuke Sotobayashi, Go Ito, Eriko Kawai, Hidehiro Nakahara, Shinya Ueda, Takeshi Toyama, Keita Saku, Yasuto Nakanishi, Hiroshi Kinoshita

**Affiliations:** ^1^ Division of Human Environment Graduate School of Human Environment Osaka Sangyo University Daito City Osaka Japan; ^2^ Department of Sport and Health Sciences Faculty of Sport and Health Sciences Osaka Sangyo University Daito City Osaka Japan; ^3^ Department of Cardiovascular Dynamics National Cerebral and Cardiovascular Center Research Institute Suita City Osaka Japan; ^4^ Department of Education Faculty of Education Osaka Seikei University Osaka City Osaka Japan; ^5^ Laboratory for Pathophysiological and Health Science RIKEN Center for Biosystems Dynamics Research Kobe City Hyogo Japan; ^6^ Graduate School of Health Sciences Morinomiya University of Medical Sciences Osaka City Osaka Japan; ^7^ Department of Physical Education Faculty of Education Gifu University Gifu City Gifu Japan; ^8^ Faculty of Medical Sciences Kyushu University Fukuoka City Fukuoka Japan; ^9^ Center for Common Education Osaka Aoyama University Minoh City Osaka Japan

**Keywords:** exercise, feedforward, heart rate, higher brain, high‐intensity, minute ventilation, performance, VO2max

## Abstract

This study aimed to investigate whether anticipatory cardiorespiratory responses vary depending on the intensity of the subsequent exercise bout, and whether anticipatory cardiorespiratory adjustments contribute importantly to enhancing exercise performance during high‐intensity exercise. Eleven healthy men were provided advance notice of the exercise intensity and a countdown to generate anticipation during 10 min prior to exercise at 0, 50, 80 or 95% maximal work‐rate (Experiment 1). A different group of subjects (*n* = 15) performed a time to exhaustion trial with or without anticipatory countdown (Experiment 2). In Experiment 1, heart rate (HR), oxygen uptake (V_O2_) and minute ventilation (V_E_) during pre‐exercise resting period increased over time and depended on the subsequent exercise intensity. Specifically, there was already a 7.4% increase in HR from more than 5 min prior to the start of exercise at 95% maximal work‐rate, followed by progressively augmented increases of 12.5% between 2 and 3 min before exercise, 24.4% between 0 and 1 min before exercise. In Experiment 2, the initial HR for the first 10 s of exercise in the task with anticipation was 11.4% larger compared to without anticipation (*p* < 0.01), and the difference in HR between the two conditions decreased in a time‐dependent manner. In contrast, the initial increases in V_O2_ and V_E_ were significantly lower in the task with anticipation than that without anticipation. The time to exhaustion during high‐intensity exercise was 14.6% longer under anticipation condition compared to no anticipation (135 ± 26 s vs. 119 ± 26 s, *p* = 0.003). In addition, the enhanced exercise performance correlated positively with increased HR response just before and immediately after exercise onset (*p* < 0.01). These results showed that anticipatory cardiorespiratory adjustments (feedforward control) via the higher brain that operate before starting exercise may play an important role in minimizing the time delay of circulatory response and enhancing performance after onset of high‐intensity exercise in man.

## Graphical abstract

What is the central question of this study? This study aimed to investigate whether anticipatory cardiorespiratory responses vary depending on the intensity of the subsequent exercise bout, and whether anticipatory cardiorespiratory adjustments contribute importantly to enhancing exercise performance during high‐intensity exercise.What is the main finding and its importance? Anticipatory cardiorespiratory control preceding exercise initiation may play an important role in minimizing the delay of circulatory response and physiological efficiency after onset of dynamic exercise, thereby enhancing maximal exercise performance during high‐intensity exercise.

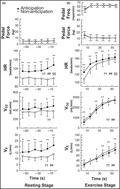

## 1 INTRODUCTION

The neural mechanisms controlling the circulatory and respiratory responses to exercise have attracted the interest of researchers since late 1800s. Central neural control, termed central command, operates in a feedforward manner and seems to be an important mechanism for rapid and appropriate matching of oxygen supply, carbon dioxide removal, and prevention of steep pH fall at the beginning of exercise (Asmussen et al., 1965; Daly & Overly., 1966; Eldridge et al., 1985; Goodwin et al., 1972; Green & Paterson, 2008; Green et al., 2007; Johansson, 1893; Krogh & Lindhard., 1913; Rowell et al., 1995; Waldrop et al., 1996; Whipp, 1977; Williamson, 2010; Williamson et al., 2006; Wood et al., 2003). Indeed, several researchers have already provided evidence for the role of central command in cardiorespiratory control, and demonstrated that the cardiorespiratory responses occur slightly before, abruptly at, or with a very short latent period after the beginning of static and dynamic exercises as well as standing (Ishii et al., 2018; Matsukawa, 2012; Matsukawa et al., 1991; Mitchell, 1990; Ninomiya et al., 1988; Secher., 2009). Previously, Smith et al. (2000) have shown that anticipatory cardiovascular responses can be produced independent of movement or posture change in free‐ranging nonhuman primates. In a human study, McArdle et al. (1967) also reported that heart rate (HR) immediately preceding the start of a running race was the highest in the shortest distance and successively lower in events of longer distance in trained runners, indicating that the anticipatory increase in HR preceding exercise onset depends on exercise training and its relationship with exercise intensity. Recently, direct measurement of cortical activity in humans and indirect assessment of oxygenation in the prefrontal cortex in humans suggests motor effort‐dependent increase of central neural drive prior to and during voluntary exercise (Green & Paterson, 2008; Green et al., 2007; Ishii et al., 2018; Williamson et al., 1999, 2006).

Mitchell (1990) reported that the relative importance of central command and exercise pressor reflex components in determining responses to exercise depends upon the type of exercise, the intensity of exercise, the time after onset of exercise, and the effectiveness of blood flow in meeting the metabolic needs of the contracting muscles. However, it remains unclear how or why centrally mediated cardiovascular responses occurring in anticipation of exercise depend on the intensity of the subsequent exercise bout. If such central neural control mechanisms play an important role in optimizing circulatory and respiratory responses to exercise, cardiorespiratory adjustments that occur in anticipation of exercise may vary as a function of the intensity of the subsequent exercise bout, and may make an important contribution to enhancing exercise performance during high‐intensity exercise, resulting in improved physiological efficiency during exercise. Indeed, Williamson (2010) suggests that if the anticipated cardiovascular adjustments (feedforward) needed to exercise were underestimated (and not corrected), exercise performance may be compromised.

The purpose of this study was to clarify the important role of feedforward control of respiration and circulation during anticipation of exercise. We performed two experiments to investigate (1) whether anticipatory cardiorespiratory responses vary depending on the intensity of the subsequent exercise bout, and (2) whether anticipatory cardiorespiratory adjustments contribute importantly to enhancing exercise performance during high‐intensity exercise.

## 2 METHODS

### 2.1 Ethical approval

The present study was approved by the Human Subjects Committee of Morinomiya University of Medical Sciences (No. 2018‐057). All procedures in the present study conformed to the ethical principles of the Declaration of Helsinki. This study was not registered in a database.

### 2.2 Participants

In Experiments 1 and 2, a total of 26 non‐smoking male subjects who enjoyed light exercise and sports once or twice a week were recruited as subjects (Table 1). All the procedures, potential risks and purposes of the study were explained thoroughly to each subject before initiation of experiments. Informed consent was obtained from each subject prior to participation in the experiment. None of the study participants were aware of the physiological mechanisms being investigated in these experiments. All subjects had no known cardiovascular or pulmonary disorders, had no history of head injury, and were not taking any prescribed medication known to influence systemic or cerebrovascular function. Prior to the experiment and after giving informed consent, each subject visited the laboratory to familiarize with the techniques and procedures. Subjects were requested to abstain from caffeine‐containing beverages for 12 h, and strenuous physical activity and alcohol for at least 24 h before the day of the experiment. To ensure familiarity with the experimental protocol, each subject performed a preliminary experiment undergoing all the experimental tasks including the maximal exercise test with advance notification of the exercise intensity, by the same procedures as used on the day of the experiment.

**TABLE 1:** Physical characteristics and maximal cardiorespiratory functions data of participants in two experiments

	Experiment 1 (*n *= 11)	Experiment 2 (*n *= 15)	*p*‐value
Age (yrs)	20.6 ± 0.5	21.1 ± 2.7	0.613
Height (cm)	173 ± 5.8	170.3 ± 3.9	0.172
Weight (kg)	71.1 ± 12.7	66.1 ± 5.2	0.188
V_O2max_ (ml/min)	3612 ± 553	3498 ± 385	0.581
V_O2max/wt_ (ml/min/kg)	51.9 ± 10.3	53.2 ± 7.0	0.539
V_Emax_ (L/min)	141.5 ± 24.5	147.0 ± 23.5	0.568
HR_max_ (beats/min)	190.6 ± 8.0	189.5 ± 7.9	0.733
WR_max_ (watt)	281.1 ± 45.5	288.2 ± 16.8	0.701

Note

Values are presented as mean ± SD. V_O2max_, maximal oxygen uptake; V_Emax_, maximal minute ventilation; HR_max_, maximal heart rate; WR_max_, maximal work rate.

### 2.3 Experimental protocols

#### 2.3.1 Maximal exercise test

The maximal oxygen uptake (V_O2max_) of each subject was assessed by a ramp‐incremental pedaling test using a computer‐controlled bicycle ergometer (AEROBIKE 75XL, Combi wellness Co.). The work‐rate was set initially at 20 watt for 3 min as warm‐up, and increased by 20 watt every minute. During exercise, the subject maintained the pedaling rate at approximately 60 rpm, and continued to pedal until he could no longer maintain the pedaling rate above 50 rpm despite strong verbal encouragement. The encouragement was actively provided when the subject's pedaling speed fell below 55 rpm. The criterion for the achievement of V_O2max_ was a plateau in O_2_ uptake (V_O2_) despite an increased work rate and respiratory exchange ratio above 1.10. Physical characteristics and maximal cardiorespiratory capacity of the experimental groups are given in Table 1. The maximal exercise performance achieved by each subject during the ramp‐incremental test was defined as 100% work‐rate (WR_max_). Based on this value, relative WRs for the subsequent tests were determined for each subject.

#### 2.3.2 Experiments

Two experiments were conducted at two different times of the year, by two different groups of subjects. Experiment 1 (*N* = 11) was conducted to examine the effects of exercise anticipation on cardiorespiratory response preceding the onset of dynamic exercise at various intensities. Experiment 2 (*N* = 15) was conducted to test whether exercise anticipation preceding onset of dynamic exercise plays an important role in the generation of cardiorespiratory responses to exercise.

**FIGURE 1:**
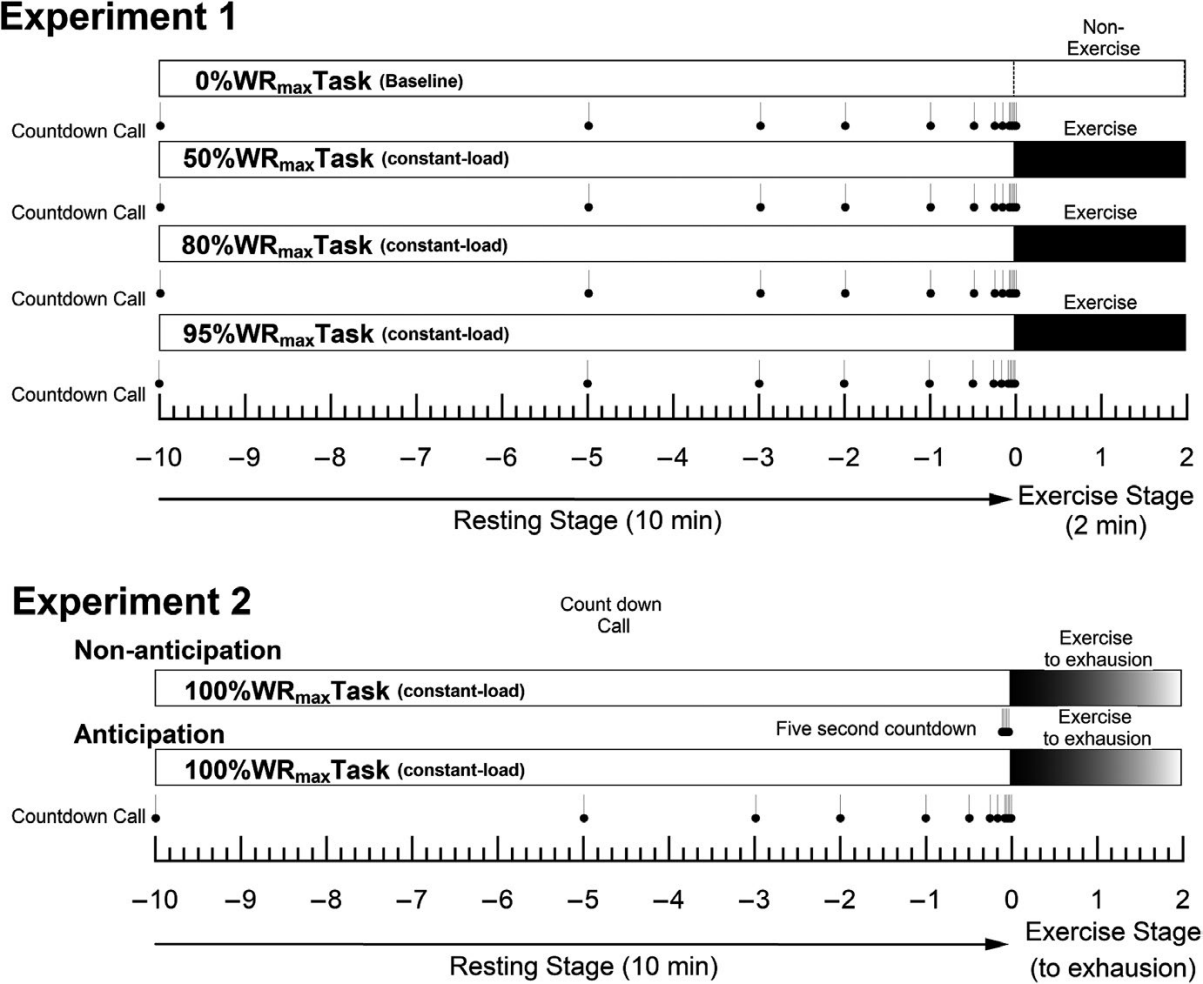
Experimental protocols

For Experiment 1, each subject performed four experimental tasks for 12 min each in a random order on different days (Figure 1). Each subject performed 3 ergometer pedaling tasks at three different predetermined %WR_max levels_: 50%, 80% and 95%, which corresponded to moderate, light heavy, and heavy workload levels. In all tasks, the subject rested while seated on a bicycle ergometer for 10 min prior to the start of exercise, and was given advance notification of the exercise intensity. During exercise, the subject maintained the pedaling rate at approximately 60 rpm regardless of the workload. In addition, a no‐pedaling task was included as control, in which the subject remained rested on the ergometer after the initial 10‐min resting period (0%WR_max_). In all these tasks, the subject was informed of the exercise onset time by the experimenter's countdown calls of the remaining time to start exercise: 10 min, 5 min, 3 min, 2 min, 1 min, 30 s, 15 s, 10 s, and every second for the last 5 s (Figure 1, Experiment 1).

For Experiment 2, each subject performed the pedaling task at 100%WR_max_ until exhaustion. This task was preceded by 10 min of resting on the ergometer seat with and without the experimenter's countdown calls. For the anticipation task, the countdown calls were given in the same manner as the exercise intensity experiment (Experiment 1). For the non‐anticipation task, the subject was told that their resting data were being measured and given no countdown call. At 5 s before the start of exercise, the subject was instructed abruptly to pedal the ergometer following a 5‐s countdown (no anticipation of exercise) (Figure 1, Experiment 2).

Under all experimental conditions, the ergometer handlebar was kept at the same height as the saddle for each individual, and the subject was instructed not to place their feet on the pedals during rest and not to release the handlebar while waiting for the start. The order of the tasks in the experiment was randomized for each subject, with an interval of at least 2 days between tasks to minimize the effect of fatigue.

### 2.4 Measurements

All studies were performed at constant room temperature between 23 and 24°C with external stimuli minimized. Respiratory and metabolic data during the experiments were recorded by an automatic breath‐by‐breath respiratory gas‐analyzing system consisting of a differential pressure transducer, sampling tube, filter, suction pump and mass spectrometer (ARCO2000‐MET, Arco System). The subject breathed through a face mask with a flow meter. Heart rate (HR) was monitored with a lead II electrocardiogram (ECG) and measured using a cardiotachometer (AT601G, Nihon Kohden) triggered by R wave on the electrocardiogram. A force transducer (TSA‐110, Takei Scientific Instruments Co. Ltd.) was attached to the left pedal of the bicycle ergometer, and the pedal reaction force and pedal frequency were measured continuously. Signals from the respiratory gas analyzer, electrocardiograph (BSM‐7201, Nihon Kohden), and force transducer attached to the pedal were synchronized on‐line using a personal computer, and displayed continuously during all experiments. Oxygen and CO_2_ measurements were calibrated using standard gas of known concentration before each test. We digitized expired flow, CO_2_ and O_2_ concentrations, and derived tidal volume, respiratory rate, minute ventilation, end‐tidal O_2_ and CO_2_ partial pressures (P_ETO2_, P_ETCO2_, respectively). Flow signals were converted to single breath data by matching to gas concentrations identified as single breaths using P_ETCO2_, after accounting for the time lag (350 ms) in gas concentration measurements. The corresponding O_2_ uptake (V_O2_), CO_2_ output (V_CO2_), and respiratory exchange ratio (RER; V_CO2_/V_O2_) for each breath were calculated from inspired‐expired gas concentration differences, and by expired ventilation (V_E_), with inspired ventilation being calculated by N_2_ correction. During each protocol, HR, O_2_, CO_2_ and flow signals were recorded continuously at 200 Hz.

### 2.5 Data analysis

For Experiments 1 and 2, mean HR, V_O2,_ V_CO2,_ V_E_, P_ETCO2_ and RER were computed for the 4‐min period from 1 to 5 min after the start of the resting stage (−9 to −4 min), and for each 1‐min period for the last 3 min of the resting stage (−3 to −2 min, −2 to −1 min, −1 to 0 min), and at the end of the exercise stage for each subject. In addition, consecutive 10‐s mean values were analyzed for the last 1 min of the resting stage, and for the first 1 min of the exercise stage to evaluate in detail the temporal differences in cardiorespiratory responses.

For Experiment 2, the peak values of the same cardiovascular variables obtained from 10‐s averaged data from exercise onset until exhaustion were used as peak cardiorespiratory function data for each individual. The time to exhaustion was defined as the interval from exercise onset until the subject could no longer maintain a pedaling frequency above 50 rpm despite strong verbal encouragement.

### 2.6 Statistical analysis

One‐way analysis of variance (ANOVA) was used to compare the means of two experimental groups. Depending on the purpose of the comparison, one‐way or two‐way ANOVA for repeated measures was conducted for each response variable using the mean data of individual subjects. A post‐hoc analysis using Tukey's multiple comparison was performed to interpret the results of significant interaction effects. Pearson product‐moment correlation coefficient (*r*) analysis was also used to examine relationship between variables. All data are presented as mean ± SD unless stated otherwise, and *p* < 0.05 was accepted as the criterion for statistical significance for all statistical analyses.

## 3 RESULTS

### 3.1 Participant characteristics

Table 1 shows the characteristics of participants and mean values of the variables describing maximal cardiorespiratory function in each study group. There were no significant differences between the two groups in all the cardiorespiratory variables.

### 3.2 Experiment 1

**FIGURE 2:**
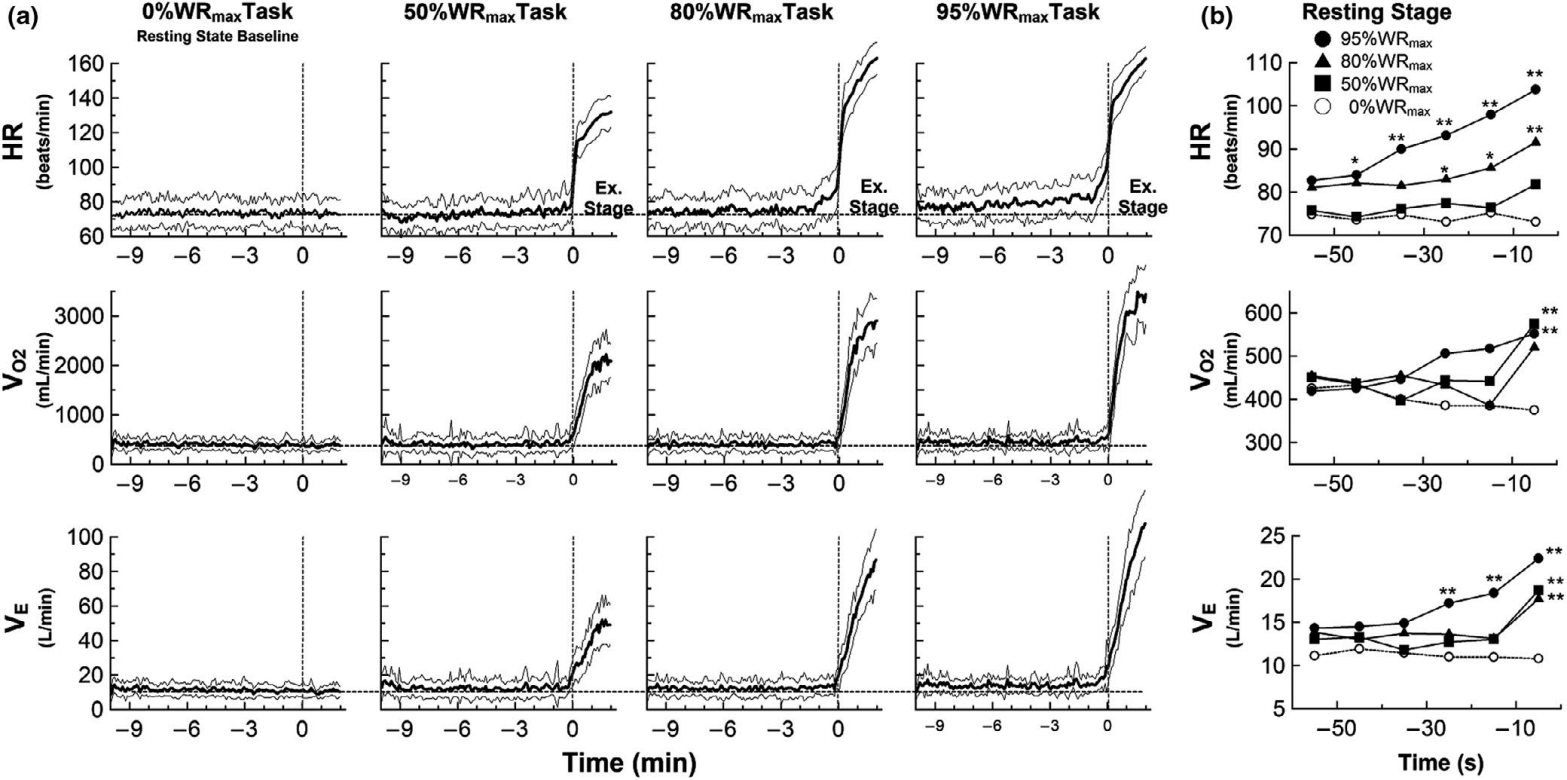
(a) Experiment 1: Time courses of mean heart rate (HR), oxygen uptake (V_O2_) and minute ventilation (V_E_) of 11 subjects during no‐exercise resting condition (0%WR_max_ task [baseline]) and exercise conditions at various intensities (50%WR_max_, 80%WR_max_ and 95%WR_max_ tasks) with advance notification of the intensity and time to start exercise10 min (time −600 s) prior to exercise onset (time 0 s). Bold lines, mean; fine lines, ±SD; horizontal dashed lines, 0%WR_max_ (baseline); vertical dashed lines, start of exercise. All cardiorespiratory variables were obtained by averaging the respective data for 1 s during each task. (b) Time‐series data of cardiorespiratory responses immediately preceding exercise onset at each task. Data are expressed as mean without SD (○, 0%WR_max_ task [baseline]; ■, 50%WR_max_ task; ▲, 80%WR_max_ task; ●, 95%WR_max_ task). The plots indicate the last 60 s of the resting stage divided into six 10‐s averaged data. Mean HR during preparatory period prior to start of exercise is higher in the 95%WR_max_ task than in the 0%WR_max_ task. Baseline shifts of HR, V_O2_ and V_E_ during anticipation period vary with time and depend on the subsequent exercise intensity. For post‐hoc analysis, *p < 0.05, **p < 0.01; significantly different versus control condition

Figure 2a shows the time courses of mean HR, V_O2_ and V_E_ for all subjects during performance of the control (0%WR_max_), and 50, 80, and 95%WR_max_ tasks. Table 2 presents the mean values of cardiorespiratory variables for the first‐half (−9 to −5 min), and the 3 consecutive pre‐exercise periods (−3 to −2 min, −2 to −1 min, −1 to 0 min) in the resting stage for all subjects and for the four test conditions. Two‐way AVOVA revealed a significant interaction effect of intensity and time for HR, V_O2_, V_CO2_, V_E_ and RER during the resting stage prior to exercise onset, indicating time‐dependent increases in cardiorespiratory variables during exercise anticipation, which also depended on the intensity of subsequent exercise (Table 2). These responses that varied over time also increased more markedly just before exercise and depended on the intensity of the subsequent exercise task. As shown in Figure 2b, compared to the control (0%WR_max_ task), there were clear time‐dependent increases in HR, V_O2_ and V_E_ as the time of exercise onset approached, and the increases in response were also clearly dependent on the expected exercise intensity; the higher the expected workload (95% and/or 80% WR_max_ tasks), the greater were the significant increase (*p* < 0.05).

**TABLE 2:** Averaged cardiorespiratory measurements in various periods of resting stage and in exercise stage for each experimental condition and comparison among different exercise intensities (Experiment 1)

Experiment 1									
	Experimental task condition	Time period of resting stage (*n *= 11)				ANOVA (*F*‐value, *p*‐value)			Exercise stage
						Main effect		Interaction	
		−9 to −5 min	−3 to −2 min	−2 to −1 min	−1 to 0 min	Condition (C)	Time (T)	C × T	End 20 s
HR (beats/min)	0%WR_max_	73.9 ± 6.8	73.7 ± 5.6	72.4 ± 7.4	74.1 ± 7.0	19.77 *p* < 0.0001	26.34 *p* < 0.0001	5.19 *p* < 0.0001	72.8 ± 7.6
	50%WR_max_	71.7 ± 6.4	73.9 ± 7.3	74.6 ± 7.0	77.0 ± 6.2				131.3 ± 9.8
	80%WR_max_	74.4 ± 7.2	75.2 ± 10.0	76.4 ± 7.1	84.3 ± 8.4				161.9 ± 9.6
	95%WR_max_	79.1 ± 7.5	82.9 ± 7.3	85.1 ± 10.7	92.2 ± 9.3				169.6 ± 8.0
V_O2_ (ml/min)	0%WR_max_	409 ± 79	380 ± 49	375 ± 67	401 ± 81	2.214 *p* = 0.107	7.371 *p* < 0.0001	2.589 *p* = 0.011	386 ± 82
	50%WR_max_	394 ± 119	412 ± 144	407 ± 110	457 ± 147				2104 ± 357
	80%WR_max_	402 ± 105	384 ± 113	397 ± 98	450 ± 128				2849 ± 450
	95%WR_max_	431 ± 70	423 ± 95	488 ± 114	477 ± 69				3263± 593
V_CO2_ (ml/min)	0%WR_max_	369 ± 91	350 ± 81	333 ± 71	360 ± 85	4.39 *p* = 0.011	13.57 *p* < 0.0001	3.26 *p* = 0.002	345 ± 80
	50%WR_max_	370± 117	395 ± 153	388 ± 113	440 ± 148				2039 ± 373
	80%WR_max_	371± 114	362 ± 116	376 ± 103	447 ± 144				3156 ± 568
	95%WR_max_	405 ± 77	396 ± 78	456 ± 129	494 ± 75				3767 ± 693
V_E_ (L/min)	0%WR_max_	11.8 ± 3.1	11.0 ± 3.0	10.6 ± 3.0	11.2 ± 2.9	6.775 *p* < 0.0001	7.345 *p* < 0.0001	3.132 *p* = 0.003	10.8 ± 3.2
	50%WR_max_	12.2 ± 4.3	12.6 ± 5.2	12.7 ± 4.5	13.8 ± 4.4				49.7 ± 12.1
	80%WR_max_	12.1 ± 3.8	12.0 ± 5.1	12.8 ± 4.6	14.3 ± 4.0				83.4 ± 16.1
	95%WR_max_	13.8 ± 2.6	13.4 ± 3.0	14.8 ± 4.0	17.1 ± 3.7				102.0 ± 19.4
P_ETCO2_ (mmHg)	0%WR_max_	40.0 ± 5.7	39.8 ± 5.3	39.9 ± 5.7	40.1 ± 5.6	5.62 *p* = 0.004	0.668 *p* = 0.578	0.578 *p* = 0.808	39.7 ± 5.8
	50%WR_max_	39.5± 5.0	40.1 ± 5.1	40.1 ± 5.3	40.3 ± 5.6				51.4 ± 6.1
	80%WR_max_	38.2 ± 5.0	38.4 ± 5.3	37.7 ± 5.5	38.7 ± 5.2				49.3 ± 6.3
	95%WR_max_	37.2± 3.2	37.0 ± 4.1	37.5 ± 4.1	37.2 ± 4.5				48.3 ± 6.0
RER	0%WR_max_	0.90 ± 0.07	0.91 ± 0.10	0.89 ± 0.05	0.89 ± 0.05	9.835 *p* < 0.0001	5.264 *p* = 0.005	2.898 *p* = 0.005	0.89 ± 0.07
	50%WR_max_	0.94 ± 0.04	0.95 ± 0.06	0.95 ± 0.05	0.96 ± 0.04				0.97 ± 0.05
	80%WR_max_	0.92 ± 0.07	0.94 ± 0.09	0.94 ± 0.06	0.99 ± 0.08				1.11 ± 0.05
	95%WR_max_	0.95 ± 0.09	0.94 ± 0.07	0.94 ± 0.07	1.04 ± 0.10				1.16 ± 0.06

Note

Vaules are presented as mean ± SD. HR, heart rate; V_E_, minute ventilation; V_O2_, oxygen consumption; V_CO2_, carbon dioxide production. P_ETCO2_, partial pressure of end‐tidal CO_2_ tensio; RER, respiratory gas exchange ratio. WR_max_, maximal work rate. 0%WR_max,_ 50%WR_max_, 80%WR_max_ and 95%WR_max_ are tasks at various intensities performed with advance notification of the exercise start time and intensity. −9 to −5 min: averaged baseline data from 1 to 5 min after start of the 10‐min resting stage (first‐half of pre‐exercise preparatory period). −1 to 0 min, −2 to −1 min, −3 to −2 min: averaged data for 0–1 min, 1–2 min and 2–3 min prior to start of exercise (second‐half of pre‐exercise preparatory period).

### 3.3 Experiment 2

**FIGURE 3:**
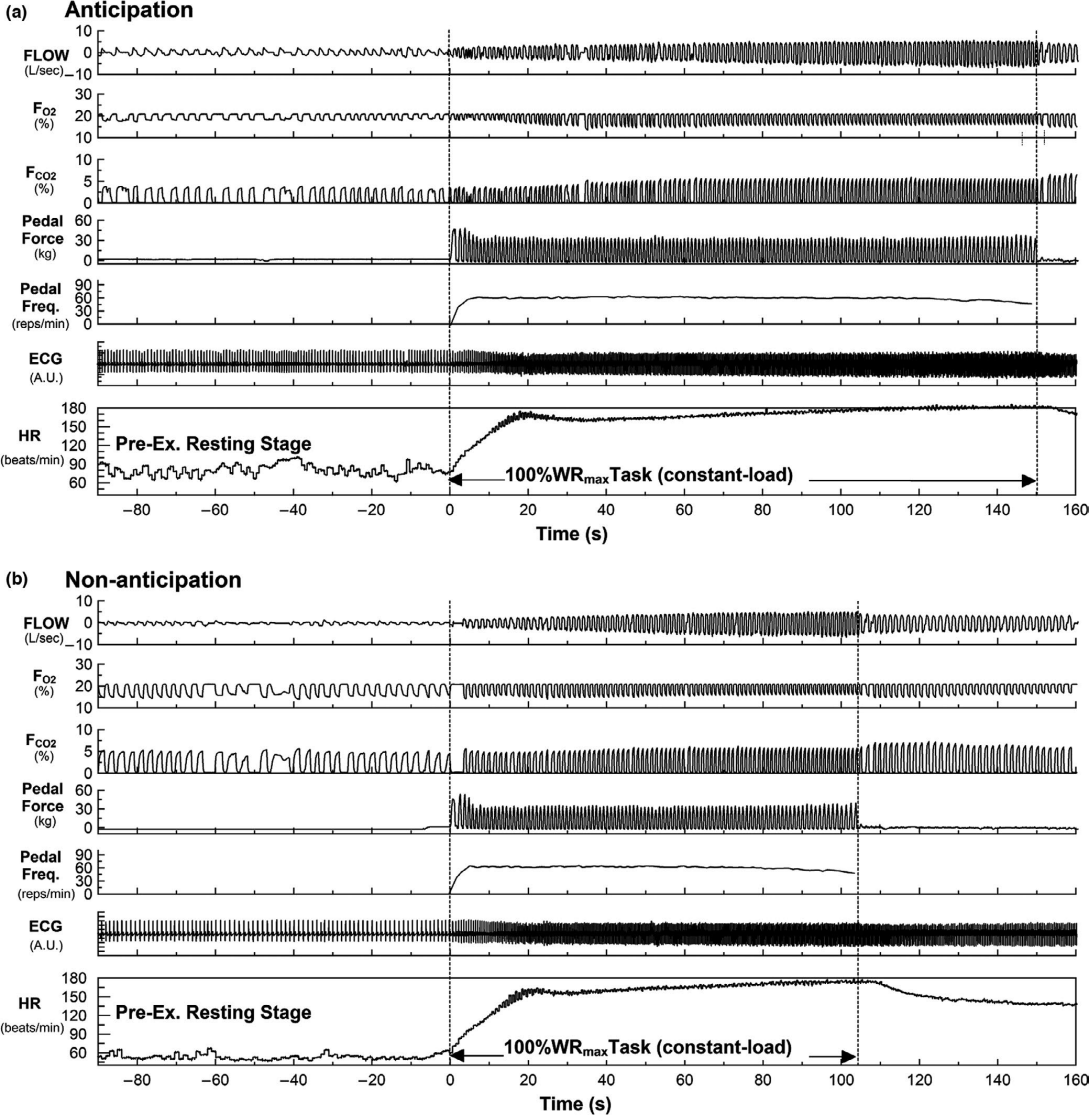
Traces of raw data in a representative subject during the 100%WR_max_ task under anticipation (a) and non‐anticipation (b) conditions (Experiment 2)

**FIGURE 4:**
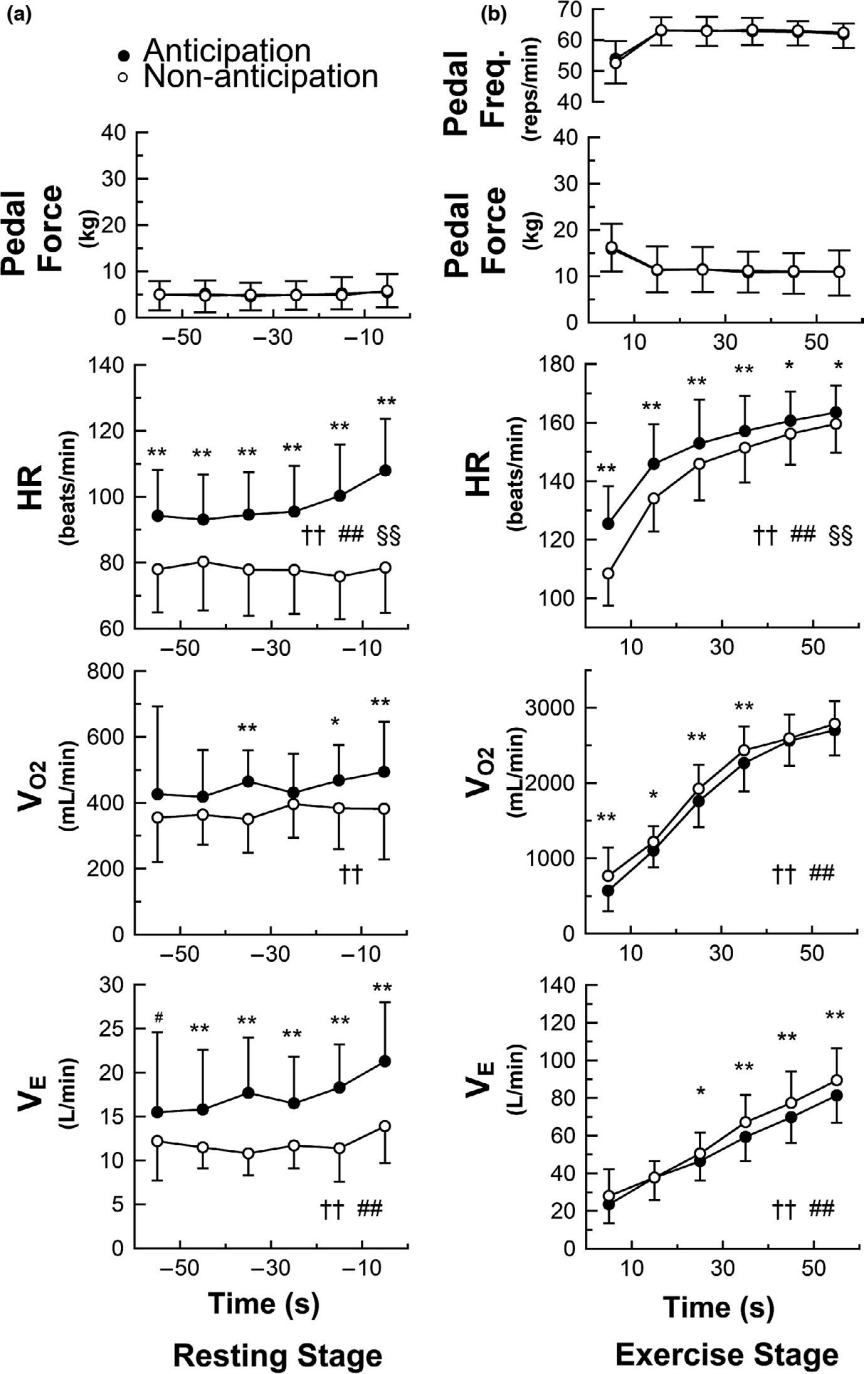
Experiment 2: Time courses of cardiorespiratory responses preceding and after exercise onset in the 100%WR_max_ task (*n* = 15) under anticipation and non‐anticipation conditions. Data are presented as mean ± SD. (●, anticipation; ○, non‐anticipation). Statistical analysis was performed using two‐way analysis of variance (ANOVA) for repeated measures. ^†^ *p* < 0.05, ^††^ *p* < 0.01; significant main effect of condition. ^#^ *p* < 0.05, ^##^ *p* < 0.01, significant main effect of time. ^§^ *p* < 0.05, ^§§^ *p* < 0.01; significant interaction effect of condition and time. For post‐hoc analysis, *p < 0.05, **p < 0.01; significantly different versus non‐anticipation conditions. During the resting stage prior to exercise onset, time‐dependent baseline shifts in cardiorespiratory variables are observed during exercise anticipation compared with non‐anticipation (a). After exercise onset, although the changes in pedal force and pedal frequency did not differ between the two conditions, the initial cardiorespiratory responses under anticipation condition are different compared to non‐anticipation condition (b)

The raw data of a representative subject performing 100%WR_max_ task under anticipation (a) and non‐anticipation (b) conditions are presented in Figure 3. The mean values for the first‐half (−9 to −5 min), and 3 consecutive pre‐exercise periods (−3 to −2 min, −2 to −1 min, −1 to 0 min) in the resting stage before performing the task under anticipation and non‐anticipation conditions are given in Table 3. Figure 4 shows the average data of the cardiorespiratory responses, pedal force, and pedal frequency over time for all subjects under anticipation and non‐anticipation conditions. During the pre‐exercise resting stage, the HR, V_O2_, and V_E_ responses were higher under anticipation condition than under non‐anticipation condition. Furthermore, these cardiorespiratory variables tended to increase with time under anticipation condition compared with non‐anticipation. As shown in Figure 4a, compared to the non‐anticipation condition (0%WR_max_ task), there was a clear time‐dependent upward trend in all the cardiorespiratory variables as the time of exercise onset approached, and the trend was also clearly dependent on the expected workload (condition). Two‐way AVOVA revealed a significant interaction effect of condition and time for HR, V_CO2_, V_E_, P_ETCO2_ and RER during the pre‐exercise resting stage, indicating time‐dependent increases in cardiorespiratory variables during the 100%WR_max_ task with exercise anticipation compared to without anticipation (Table 3).

**TABLE 3:** Averaged cardiorespiratory measurements in various periods of resting stage and in exercise stage for each experimental condition and comparison between anticipation and non‐anticipation (Experiment 2)

Experiment 2 (100%W_Rmax_)									
	Experimental task condition	Time period of resting stage (*n *= 15)				ANOVA (*F*‐value, *p*‐value)			Exercise stage
						Main effect		Interaction	
		−9 to −5 min	−3 to −2 min	−2 to −1 min	−1 to 0 min	Condition (C)	Time (T)	C × T	Peak‐value
HR (beats/min)	Non‐anticipation	73.6 ± 14.8	75.3 ± 15.0	75.7 ± 14.3	78.1 ± 14.1	41.98 *p* < 0.0001	58.20 *p* < 0.0001	14.85 *p* < 0.0001	173.3 ± 10.1
	Anticipation	81.0 ± 11.8	84.8 ± 12.6	88.2 ± 14.1	97.6 ± 15.9				177.5 ± 8.0
V_O2_ (mL/min)	Non‐anticipation	362 ± 132	334 ± 125	341 ± 111	372 ± 153	15.81 *p* = 0.001	12.20 *p* < 0.0001	2.72 *p* = 0.057	3164 ± 388
	Anticipation	362 ± 146	364 ± 149	394 ± 164	450 ± 191				3172 ± 424
V_CO2_ (mL/min)	Non‐anticipation	324 ± 121	305 ± 112	312 ± 102	338 ± 136	24.93 *p* < 0.0001	14.86 *p* < 0.0001	5.81 *p* = 0.002	3986 ± 541
	Anticipation	337 ± 140	360 ± 148	394 ± 168	481 ± 215				4042 ± 522
V_E_ (L/min)	Non‐anticipation	11.1 ± 3.9	10.7 ± 3.6	10.9 ± 3.4	11.9 ± 4.5	21.70 *p* < 0.0001	18.53 *p* < 0.0001	7.14 *p* = 0.001	139.5 ± 27.9
	Anticipation	11.8 ± 4.7	12.4 ± 4.9	13.7 ± 6.0	17.5 ± 7.8				146.1 ± 23.7
P_ETCO2_ (mmHg)	Non‐anticipation	38.2 ± 3.2	36.8 ± 3.9	36.7 ± 4.2	37.8 ± 4.3	0.164 *p* = 0.692	0.331 *p* = 0.803	4.75 *p* = 0.006	47.1 ± 4.7
	Anticipation	36.5 ± 5.6	38.1 ± 3.9	38.0± 4.7	37.3 ± 5.0				47.9 ± 3.6
RER	Non‐anticipation	0.90 ± 0.06	0.92 ± 0.09	0.92 ± 0.09	0.92 ± 0.07	13.73 *p* = 0.002	7.27 *p* < 0.0001	7.42 *p* < 0.0001	1.29 ± 0.08
	Anticipation	0.93 ± 0.08	0.99 ± 0.11	1.00 ± 0.13	1.06 ± 0.13				1.30 ± 0.06

Note

Vaules are presented as mean ± SD. HR, heart rate; V_E_, minute ventilation; V_O2_, oxygen consumption; V_CO2_, carbon dioxide production. P_ETCO2_, partial pressure of end‐tidal CO_2_ tensio; RER, respiratory gas exchange ratio. −9 to −5 min: averaged baseline data from 1 to 5 min after start of the 10‐min resting stage (first‐half of pre‐exercise preparatory period). −1 to 0 min, −2 to −1 min, −3 to −2 min: averaged data for 0–1 min, 1–2 min and 2–3 min prior to start of exercise (second‐half of pre‐exercise preparatory period).

After the onset of exercise in the 100%WR_max_ task, remarkable increases in HR, V_O2_ and V_E_ were also shown and the responses augmented under both anticipation and non‐anticipation conditions. Although the changes in pedal force and pedal frequency did not differ between the two conditions after exercise onset in the 100%WR_max_ task. The HR increase after exercise onset was higher while increases of V_O2_ and V_E_ were lower under anticipation condition compared to non‐anticipation condition. The differences tended to decrease depending on the time (Figure 4b). Two‐way AVOVA revealed a significant interaction effect of condition and time for HR and RER during 100%WR_max_ task (*p* < 0.0001), indicating time‐dependent upward shift in baseline cardiorespiratory variables during exercise anticipation compared with non‐anticipation.

**FIGURE 5:**
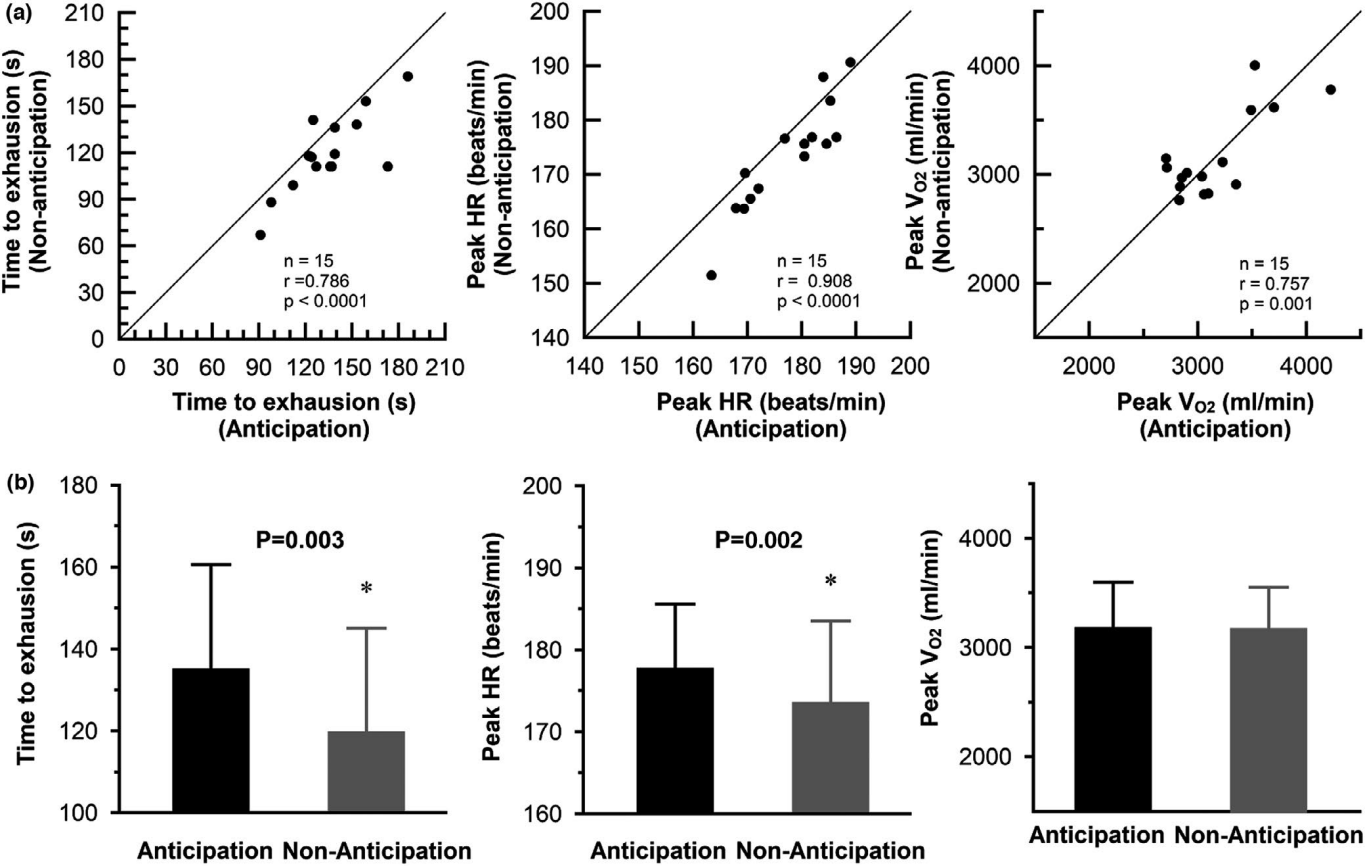
Anticipation versus non‐anticipation scatter‐plots (a) and bar charts of averaged data (b) for time to exhaustion, peak HR and peakV_O2_ during exercise to exhaustion at 100%WR_max_ in 15 subjects (Experiment 2). The points below the identity line represent decreased measurement data in the non‐anticipation condition compared with anticipation condition in individual participants. *; significantly different versus non‐anticipation conditions. In B, data are presented as mean ± SD. Black bar, anticipation; Grey bar, non‐anticipation; Solid line, identity line. Mean time to exhaustion and peak HR under anticipation condition are 14.6 ± 15.8% (*p* = 0.003) and 2.4 ± 2.5% (*p* = 0.002), respectively, higher than those under non‐anticipation condition. There is no significant difference in _Peak_V_O2_ between the two conditions

Figure 5a shows the non‐anticipation versus anticipation scatter‐plots for time to exhaustion, peak HR and peak V_O2_ from exercise onset to exhaustion in the 100%WR_max_ task. A significant positive correlation between anticipation and non‐anticipation conditions was found for all variables (time to exhaustion, *r* = 0.786, *p* = 0.005; peak HR, *r* = 0.908, *p* < 0.0001; peak V_O2_, *r* = 0.757, *p* = 0.0011). All but one subject showed reduced exercise performance under non‐anticipation condition compared with anticipation condition. On average, time to exhaustion was 14.6 ± 15.8% longer (*p* = 0.003) and peak HR was 2.4 ± 2.5% higher (*p* = 0.002) under anticipation condition compared with non‐anticipation condition. There were no significant differences in peak V_O2_, V_CO2_, V_E_, P_ETCO2_ and RER between the two conditions (Figure 5b and Table 3).

**FIGURE 6:**
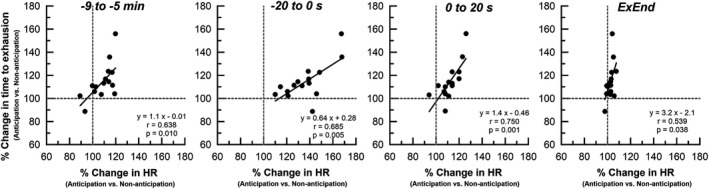
Relation between the percentage difference (anticipation/non‐anticipation) in time to exhaustion and percentage difference in HR response. Percent difference in time to exhaustion correlates significantly with percentage difference in HR for the first‐half (−9 to −5 min) (*r* = 0.638, *p* = 0.010) and the last 20 s (−20 to 0 s) (*r* = 0.685, *p* = 0.005) of pre‐exercise resting stage, the first 20 s (0 to 20 s) of exercise stage (*r* = 0.750, *p* = 0.001), and the last 10 s before exhaustion (ExEnd) (*r* = 0.539, *p* = 0.038). Straight line, linear regression

Figure 6 shows the relation between the percent differences (anticipation/non‐anticipation) in time to exhaustion and HR response. Percent difference in time to exhaustion correlated positively with percent difference in HR for the first‐half (−9 to −5 min) (*r* = 0.638, *p* = 0.010) and the last 20 s (−20 to 0 s) (*r* = 0.685, *p* = 0.005) of pre‐exercise resting stage, the first 20 s of exercise stage (0 to 20 s) (*r* = 0.750, *p* = 0.001), and the last 10 s before exhaustion (ExEnd) (*r* = 0.539, *p* = 0.038).

## 4 DISCUSSION

This experimental study provides new evidence for feedforward‐mediated cardiorespiratory responses that can be observed before the onset of exercise at different exercise intensities, providing insight into the importance and the physiological significance of central modulation of cardiorespiratory responses to exercise anticipation in man. The main findings of the study are as follows.

1. When a subject was given advance notification of the starting time and intensity of exercise to be performed (anticipation condition), the HR, V_O2_ and V_E_ during the pre‐exercise resting period increased over time and was dependent on the subsequent exercise intensity.
2. The initial increases in HR responses after starting high‐intensity exercise in subjects under anticipation condition were larger than those in subjects under non‐anticipation condition, and the differences in responses between the two conditions decreased depending on exercise time.
3. The time to exhaustion during high‐intensity exercise was 14.6% longer under anticipation condition compared to no anticipation. In addition, the enhanced exercise performance correlated positively with increased HR response just before and immediately after exercise onset (*p* < 0.01).

### 4.1 Effect of exercise anticipation on cardiorespiratory dynamics prior to exercise onset

As originally suggested by Krogh and Lindhard (1913), the existence of central neural mechanisms controlling the circulatory and respiratory responses to exercise has been confirmed by both animal and human studies, providing evidence that feedforward mediated physiological responses operate based on neural activities in the motor cortex or the hypothalamus, and that cardiorespiratory responses occur slightly before, abruptly at, or with a very short latent period (1 s) after the beginning of static and dynamic exercises, standing, or eating behavior (Cechetto, 2014; Daly & Overly., 1966; Eldridge et al., 1985; Ishii et al., 2018; Matsukawa, 2012; Matsukawa et al., 1991; Mitchell, 1990; Ninomiya et al., 1988; Toska & Eriksen., 1994; Waldrop et al., 1996; Williamson, 2010; Williamson et al., 1999, 2006). In human studies, Callister et al. (1994) reported that the increases in HR and blood pressure during the preparation phase averaged 7 beats/min and 5–6 mmHg, respectively, and were not related to power output (averaging 11%–66% of peak workload). On the other hand, McArdle et al. (1967) found that HR immediately preceding the start of track running increased, and the magnitude of increase in anticipatory HR was greater in runners trained for sprint events (67–148 beats/min) than in those trained for endurance running (59–108 beats/min). Thus, the results of HR response during the anticipation period prior to onset of exercise at different intensities observed in this study are consistent with the results of previous human studies. In addition, we found that the change in HR response during the anticipation period prior to exercise onset increased over time and depended on the intensity of subsequent exercise, suggesting a role of neural control mechanism in the higher brain. Specifically, there was already a 7.4% increase in HR (from 74 to 79 beats/min) from more than 5 min prior to the start of high‐intensity exercise (95%WR_max_ task), followed by progressively augmented increases of 12.5% between 2 and 3 minutes before exercise, 24.4% between 0 and 1 minute before exercise (Table 2), and 41.9% during just 10 seconds before exercise (Figure 2a,b). Similar changes in HR responses prior to the start of high‐intensity exercise (100%WR_max_) were also observed under anticipation condition in Experiment 2 (Figure 4a, Table 3). More surprisingly, we also found that not only pre‐exercise HR but also time‐dependent baseline shifts of V_O2_ and V_E_ varied depending on the intensity of subsequent exercise (Figure 2, Table 2). Very similar changes were observed under anticipation condition in Experiment 2 (100%WR_max_) (Figure 4a, Table 3). Although the underlying mechanisms by which cardiorespiratory and metabolic changes occur during exercise anticipation have not been elucidated, increases in V_O2_ during anticipation appear related to the elevated V_E_ (i.e., increased work of breathing) (Otis, 1954). Previous studies have shown that cortical activity associated with exercise anticipation leads to an increase in intensity and/or time dependence of cardiorespiratory and metabolic responses during the anticipation period, suggesting the existence of exercise experience‐based learning mechanism (Wood et al., 2003). Moreover, recently, Fisher et al. (2015) reported that anticipation of exercise blunted carotid baroreflex mediated HR responses, but while a clear blunting of HR response was observed in the first two trials, habituation of the response was found in later trials. Their finding may support the involvement of cortical control of the cardiovascular system in exercise anticipation, and may explain the existence of exercise experience‐based learning mechanism.

### 4.2 Effect of exercise anticipation on cardiorespiratory dynamics after exercise onset

In Experiment 2, after the onset of exercise at 100%WR_max_, initial HR for the first 10 s of exercise increased 11.4% under anticipation condition compared to non‐anticipation condition (125 bpm vs. 110 bpm). Furthermore, a difference in HR increase between the two conditions remained detectable until 50 s after initiation of exercise (Figure 4b). Consequently, feedforward control by higher brain centers during exercise preparation may play an important physiological role in achieving rapid and enhanced cardiac response to exercise, especially strenuous exercise (Figure 4b). A number of studies have shown that the initial HR and V_E_ responses after exercise onset are very rapid, and that some variables may even increase in anticipation of exercise. Mitchell (1990) proposed that central command from the higher brain controls cardiorespiratory functions after exercise onset, and can stimulate a centrally generated command signal that elicits a parallel activation of motor and cardiovascular centers. Given the limited anaerobic capacity, such regulatory mechanisms are important for rapid delivery of oxygen to the active muscles (Secher, 2009). Taken together, previous findings and the present results indicate that the feedforward mechanism contributes significantly to the acceleration of HR increase after the start of strenuous exercise, and is already functioning before exercise is started. In other words, it may be speculated that activation of the central command or feedback control mechanism after exercise onset is insufficient to provide rapid and sufficient increase in blood flow to meet the metabolic demands of the working muscles once vigorous exercise is started, and supplementation of preliminary feedforward control prior to exercise onset can dramatically improve physiological efficiency during exercise (Williamson, 2010). Indeed, our experimental results corroborate this hypothesis. The present findings support the concept of central circulatory and respiratory control that has been discussed by many researchers in the past.

### 4.3 Effect of exercise anticipation on metabolic measurement and maximal exercise performance

The most interesting finding of the present study is that the time to exhaustion during vigorous exercise was 14.6 ± 15.8% longer under anticipation condition compared to no anticipation (134.7 ± 25.7 s versus 119.3 ± 25.5 s; *p* = 0.003) (Figure 5). In addition to the enhanced exercise performance correlating with increased HR response just before and immediately after exercise onset, there was already a baseline shift in HR immediately after subjects were informed of the intensity and time to start exercise, and the rate of HR change strongly affected performance (Figure 6, Table 3). These findings seem to support our hypothesis that an intensity‐ and time‐dependent anticipatory cardiorespiratory response to exercise reduces the delay of circulatory response and O_2_ delivery to active muscle tissue after onset of strenuous exercise, thereby reducing the reliance on or buffering the use of anaerobic energetic pathways and improving maximal exercise performance compared with non‐anticipation condition. According to the above hypothesis, the V_O2_ response at the onset of exercise would increase more rapidly under the anticipation condition. Contrary to this expectation, however, the initial V_O2_ increase for 1 min after exercise onset under the anticipation condition was 9% smaller than that under non‐anticipation condition (Figure 4b). The physiological mechanism responsible for the difference in V_O2_ dynamics after exercise onset between anticipation and no anticipation conditions are unclear. However, it is unlikely that decrease in oxygen supply after exercise onset with anticipation causes this phenomenon, because peak V_O2_ remained unchanged between the two conditions. On the other hand, Chin et al. (2013) examined V_O2_ and leg blood flow kinetics at the onset of moderate‐intensity exercise during hyperventilation with and without associated hypocapnic alkalosis, and found that hypocapnia/alkalosis was responsible for the slower leg blood flow response, but hyperventilation per se had a role to play in the slower V_O2_ kinetics. Therefore, it does seem that a slower V_O2_ kinetics secondary to hyperventilation may have been apparent. In this investigation, we cannot provide our thoughts regarding how this slower V_O2_ onset is compatible with improved maximal exercise performance. However, taken together, above findings suggest that lower V_E_ response and subsequent reduced work of breathing during exercise in the anticipatory condition may be the mechanism underlying several phenomena observed in this study. Indeed, Harms et al. (2000) reported that the work of breathing normally incurred during sustained heavy‐intensity exercise had a significant influence on exercise performance, and they showed approximately 14% increase in time to exhaustion in respiratory muscle unloading trials compared with control. As another mechanism, it may be important to consider the effects of anticipatory input (feedforward) on the perception of effort/exertion and somatosensory performance. Being able to mentally prepare for a sprint is expected to improve performance, and knowledge of exertional cues can impact perception of effort (Williamson, 2010); in turn, perception of effort can impact central neural drive (Amann & Dempsey, 2008). We speculate that there may be differences in neuromuscular recruitment and efficiency in early exercise between anticipated and unanticipated exercise. In the future, experimental studies incorporating controlled breathing as well as measurements of electromyography activity of the leg muscles, brain cortical activity and other metabolic parameters such as blood lactate and catecholamine during the same experimental paradigm will provide more insight into these issues. Further research on the cardiorespiratory control mechanisms in the higher brain before and during exercise is necessary for elucidation of the role of central neural control in the autonomic nervous system and the limiting factors of maximal exercise performance.

### 4.4 Physiological Implication

Having experimentally verified and interpreted the mechanisms of cardiorespiratory dynamics at the start of high‐intensity exercise through the present study, the mental and physiological conditions of the subjects preparing to start exercise should be considered. In other words, the time series of changes in intensity‐dependent circulatory and respiratory responses during the pre‐exercise anticipatory period provide a large number of learning elements, and the effects significantly impact cardiorespiratory responses during exercise. In future research on high‐intensity exercise, attention should be paid to the subject's exercise experience and the characteristics of the sport, especially when selecting the type and intensity of exercise. The present findings are also expected to contribute to psychophysiological study of preconditioning, which may be involved in determining the maximum exercise performance of top athletes. Although various mechanisms aiming to improve performance have been proposed in previous reports, there is a possibility that the central regulatory mechanism through higher brain function during anticipation before exercise onset may also be a factor. In patients with hypertension and heart failure, exercise pressor reflex overactivity has been suggested to reduce exercise tolerance and increase the risk of adverse cardiac events and stroke during exercise (Mitchell & Smith, 2008). Thus, understanding the relative importance of central neural drive and exercise pressor reflex components in determining cardiorespiratory responses to exercise has clinical importance. Finally, this study poses new research questions on interesting physiological aspects that remain to be addressed, such as neuromuscular mobilization, hyperventilation, perception of effort and how it impacts central drive, and flight‐or‐fight and alerting/defense responses. In future studies, it is well worth considering whether higher brain mechanisms including learning and memory play an important role in matching cardiorespiratory responses with metabolic rates during pre‐exercise and/or exercise period.

### 4.5 Study limitation

Since cortical and systemic skeletal muscle activities were not measured under all experimental conditions, it is unclear whether the observed changes in cardiorespiratory and metabolic responses are mediated by changes in higher brain activity. In addition, the experimenter's countdown calls and anticipation of the intensity of the subsequent exercise may cause conscious and unconscious preparatory motions that would affect cardiorespiratory and metabolic responses. To avoid these confounding effects, subjects were strongly instructed not to change their resting posture, to place their feet on the pedals, and not to release the handlebar of the bicycle ergometer without contract muscles isometrically (such as gripping the handles, squeezing the legs) while waiting to start. In addition, a force transducer was attached to the left pedal of the bicycle ergometer, and the pedal reaction force was continuously measured. We also confirmed that there was no change in body movement by visual observation. Therefore, the observed changes in cardiorespiratory and metabolic responses are mostly likely mediated by central neural drive during anticipation of exercise. In this study, the heart rate was the only index of the circulatory system evaluated. To understand the physiological mechanisms that determine the dynamics of circulatory and respiratory responses during all experimental conditions, it is necessary to measure the time course of cardiac output and blood pressure and to evaluate and validate the role of central neural regulation on the systemic circulation including metabolic variables, since acceleration of heart rate itself has no power to increase blood flow. It is well known that age and gender each have a significant impact on the cardiac response to exhaustive upright cycle exercise. For example, previous study indicates a differential regulation of cardiovascular function and exercise efficiency between sexes during constant‐load submaximal exercise (Charkoudian & Joyner, 2004; Fleg et al., 1995; Wheatley et al., 2014). Therefore, female and older participants were not included in this study because the possibility that age or gender differences may affect cardiorespiratory adjustments made in anticipation of exercise cannot be completely ruled out.

## 5 CONCLUSION

Anticipatory cardiorespiratory control preceding exercise initiation may play an important role in minimizing the delay of circulatory response and physiological efficiency after onset of dynamic exercise, thereby enhancing maximal exercise performance during high‐intensity exercise in man.

## CONFLICT OF INTEREST

No conflicts of interest, financial or otherwise, are declared by the authors.

## AUTHOR CONTRIBUTION

T.M. and H.K.: conceived and designed the research; D.S., G.I., E.K., H.N., S.U., T.T., K.S., Y.N. and T.M.: performed experiments; T.M. and D.S.: analyzed data; T.M. and H.K.: interpreted results of experiments; T.M.: prepared figures; T.M. and H.K.: drafted the manuscript; all authors edited and revised manuscript. All authors have read and approved the final version of this manuscript and agree to be accountable for all aspects of the work in ensuring that questions related to the accuracy or integrity of any part of the work are appropriately investigated and resolved. All persons designated as authors qualify for authorship, and all those who qualify for authorship are listed.
